# Pineal Calcification, Melatonin Production, Aging, Associated Health Consequences and Rejuvenation of the Pineal Gland

**DOI:** 10.3390/molecules23020301

**Published:** 2018-01-31

**Authors:** Dun Xian Tan, Bing Xu, Xinjia Zhou, Russel J. Reiter

**Affiliations:** Department of Cell Systems & Anatomy, UT Health San Antonio, San Antonio, TX 78229, USA; doctxu@126.com (B.X.); ZhouX4@uthscsa.edu (X.Z.)

**Keywords:** pineal gland, calcification, melatonin, aging, neurodegenerative diseases, rejuvenation

## Abstract

The pineal gland is a unique organ that synthesizes melatonin as the signaling molecule of natural photoperiodic environment and as a potent neuronal protective antioxidant. An intact and functional pineal gland is necessary for preserving optimal human health. Unfortunately, this gland has the highest calcification rate among all organs and tissues of the human body. Pineal calcification jeopardizes melatonin’s synthetic capacity and is associated with a variety of neuronal diseases. In the current review, we summarized the potential mechanisms of how this process may occur under pathological conditions or during aging. We hypothesized that pineal calcification is an active process and resembles in some respects of bone formation. The mesenchymal stem cells and melatonin participate in this process. Finally, we suggest that preservation of pineal health can be achieved by retarding its premature calcification or even rejuvenating the calcified gland.

## 1. Introduction

Pineal gland is a unique organ which is localized in the geometric center of the human brain. Its size is individually variable and the average weight of pineal gland in human is around 150 mg [1], the size of a soybean. Pineal glands are present in all vertebrates [2]. Pineal-like organs are also found in non-vertebrate organisms such as insects [3,4,5]. It appears that the sizes of pineal glands in vertebrates are somehow associated with survival in their particular environments and their geographical locations. The more harsh (colder) their habitant, the larger their pineal glands are. A general rule is that the pineal gland increases in size in vertebrates from south to north or from the equator to the poles [6]. It is unknown whether if the same species moved to a different environment this would cause a change in the size of their pineal gland.

It was reported that several physiological or pathological conditions indeed alter the morphology of the pineal glands. For example, the pineal gland of obese individuals is usually significantly smaller than that in a lean subject [7]. The pineal volume is also significantly reduced in patients with primary insomnia compared to healthy controls and further studies are needed to clarify whether low pineal volume is the basis or a consequence of a functional sleep disorder [8]. These observations indicate that the phenotype of the pineal gland may be changeable by health status or by environmental factors, even in humans. The largest pineal gland was recorded in new born South Pole seals; it occupies one third of their entire brain [9,10]. The pineal size decreases as they grow. Even in the adult seal, however, the pineal gland is considerably large and its weight can reach up to approximately 4000 mg, 27 times larger than that of a human. This huge pineal gland is attributed to the harsh survival environments these animals experience [11].

The human pineal gland has been recognized for more than 2000 years. The father of anatomy, the Greek anatomist, Herophilus (325–280 BC), described the pineal gland as a valve of animal memory. René Descartes (1596–1650), a French philosopher, mathematician, and scientist, regarded the pineal gland as the principal seat of the soul and the place in which all thoughts are formed. A real biological function of pineal gland was not uncovered until 1958 [12], that is, this gland is a secretory organ which mainly produces and releases a chemical, called melatonin, into the blood circulation and into the cerebrospinal fluid (CSF). In addition, it also produces some peptides [13,14] and other methylated molecules, for example, *N*,*N*-dimethyltryptamine (DMT or *N*,*N*-DMT) [15,16], a potent psychedelic. This chemical was suggested to be exclusively generated by the pineal gland at birth, during dreaming, and/or near death to produce “out of body” experiences [17]. However, the exact biological consequences (if any) of these substances remain to be clarified. Recently, it was reported that pineal gland is an important organ to synthesize neurosteroids from cholesterol. These neurosteroids include testosterone (T), 5α- and 5β-dihydrotestosterone (5α- and 5β-DHT), 7α-hydroxypregnenolone (7α-OH PREG) and estradiol-17β (E2). The machinery for synthesis of these steroids has been identified in the gland. 7α-OH PREG is the major neurosteroid synthesized by the pineal gland. Its synthesis and release from gland exhibits a circadian rhythm and it is regulates the locomote activities of some vertebrates, especially in birds [18]. These observations opened a new avenue for functional research on pineal gland; the observations require further confirmation.

The most widely accepted concept is that melatonin is the recognized major product of the pineal gland. Melatonin is the derivative of tryptophan. It was first isolated from the pineal gland of the cow and it was initially classified as a neuroendocrine-hormone [19]. Subsequently, it was discovered that retina [20,21] and Harderian gland [20,22,23,24] also produced melatonin. Recently, it has been found that almost all organs, tissues and cells tested have the ability to synthesize melatonin using the same pathway and enzymes the pineal uses [25,26]. These include, but not limited to, skin [27], lens [28], ciliary body [29,30], gut [31,32], testis [33], ovary [34,35], uterus [36], bone marrow [37,38], placenta [39,40], oocytes [41], red blood cells [42], plantlets [43], lymphocytes [44], astrocytes, glia cells [45], mast cells [46] and neurons [47]. Not only melatonin but also the melatonin biosynthetic machinery including mRNA and proteins of arylalkylamine *N*-acetyltransferase (*AANAT*) and/or *N*-acetyl-serotonin methyltransferase (*ASMT*) [formerly hydroxyindole*O*-methyltransferase (*HIOMT*)] have been identified in these organs, tissues and cells. It was calculated that the amounts of extrapineal derived melatonin is much greater than that produced by the pineal [48]. However, the extra pineal-derived melatonin cannot replace/compensate for the role played by the pineal-derived melatonin in terms of circadian rhythm regulation. As we know pineal melatonin exhibits a circadian rhythm in circulation and in the CSF with a secretory peak at night and low level during the day [19]; thus, the primary function of the pineal-derived melatonin is as a chemical signal of darkness for vertebrates [49]. This melatonin signal helps the animals to cope with the light/dark circadian changes to synchronize their daily physiological activities (feeding, metabolism, reproduction, sleep, etc.).

For the photoperiod sensitive reproductive animals, the melatonin signal regulates their reproductive activities to guide them to give birth during the right seasons [50]. Interestingly, even low ranking species that lack a pineal gland, for example, marine zooplankton, also exhibit a melatonin circadian rhythm which is responsible for their daily physiological activities [51]. While, the extrapineal melatonin in vertebrates does not contribute to the melatonin circadian rhythm and it does not serve as the chemical signal of darkness since pinealectomy in animals distinguishes this rhythm [52,53,54]. This was further confirmed by the recent discovery that the expressions of *AANAT* and *ASMT* are present in mitochondria of both pinealocytes and neuron cells and their mitochondria synthesized melatonin. However, the expressions of *AANAT* and *ASMT* exhibit a circadian rhythm that matched the fluctuation in melatonin levels only in the mitochondria of pineal gland while this rhythm was absent in the mitochondria of neuronal cells [55]. Thus, the primary function of extrapineal melatonin (except for the retina; retinas not only possess an internal melatonin rhythm [56,57]; retinal melatonin might participate in melatonin circadian rhythm of the general circulation in some species [58,59,60]) is to serve as an antioxidant, autocoid, paracoid and tissue factor locally [49,61].

In addition to synthesizing the “signaling-melatonin” which differs from extrapineal melatonin, the pineal gland also participates in the CSF production and recycling. The blood filtration rate of this gland is comparable to the kidney [62]; this is, far more than its metabolic requirement. It was hypothesized that pineal gland may function like the kidney as a blood filter to generate CSF; this is similar to the function of choroid plexus to recycle the CSF [63]. Pineal gland and choroid plexus share a similar vasculature structure with the abundance of the vasculature spaces and fenestrated capillaries. A direct morphological connection between pineal gland and choroid plexus has been reported in birds [64]. The functional and vascular structural similarities may explain the high calcification rates of both structures [65,66].

The calcium deposits in the pineal gland were recognized several decades ago in vertebrates [67,68]. Some researchers believe that pineal calcification was associated with certain endocrine diseases such as schizophrenia, and mammary carcinoma [69,70,71,72,73,74,75]. Others feel that it is a natural process and has no consequences for human physiopathology since this process occurs early in childhood [76] and it also may not impact the melatonin synthetic ability of the gland in some animals [77,78]. Recently, additional studies have shown that pineal calcification indeed jeopardizes the melatonin production in humans and it seems to have a direct influence on neurodegenerative diseases and aging [79,80,81]. This review summarizes the current developments in the field and also provides opinions and comments on pineal physiology and pineal gland calcification (PGC).

## 2. Pineal Gland and the Melatonin Circadian Rhythm

**Figure 1:**
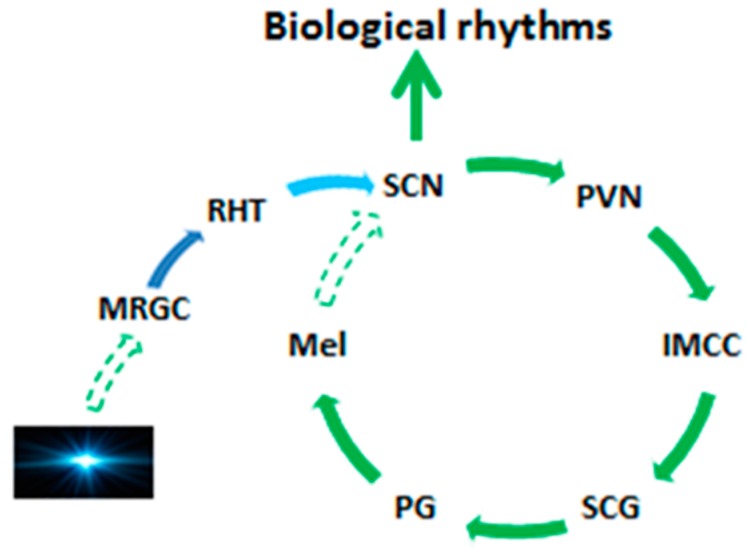
Illustration of the SCN-melatonin loop. Solid arrows indicate the neuronal connections and the direction of neuronal projections. Dash arrows indicate the input signals. SCN is the master clock which determines the biological rhythms as well as the melatonin circadian rhythm. Its intrinsic circadian interval is longer than 24 h. The natural photoperiod (photos) serves as an input signal to entrain melatonin circadian rhythm to 24 h; in turn, melatonin functions as a signal of photoperiod to re-entrain the biological rhythm of SCN to 24 h. MRGC: melanopsin-containing retinal ganglion cells; RHT: retino-hypothalamic tract; SCN: suprachasmatic nucleus; PVN: paraventricular nucleus; IMCC: Intermediolateral cell column; SCG: sympathetic cervical ganglion; PG: pineal gland. Mel: melatonin.

The pineal gland is situated in the geometric center of the human brain and it is directly connected to the third ventricle; it is classified as a circumventricular organ (CVO) and participates in the biological rhythm regulation in vertebrates. Herein, we refer to the structures which regulate biorhythms as the suprachiasmatic nucleus (SCN)-melatonin loop. This loop includes melanopsin-containing retinal ganglion cells (MRGC), retino-hypothalamic tract (RHT), SCN, paraventricular nucleus (PVN), Intermediolateral cell column, sympathetic cervical ganglia (SCG), the pineal gland, melatonin rhythm which feedback impacts the SCN (Figure 1).

Any defect of the loop results in a diminished melatonin circadian rhythm and the disturbance of chronobiology. For example, SCN or PVN lesions [82,83,84], blockade of the cervical ganglia [85,86] or pinealectomy [52,53] is always accompanied by the loss of the melatonin rhythm in vertebrates. This loop is important to regulate the biological rhythms of vertebrates. SCN is believed to be the master clock or the pacemaker [87]. This pacemaker has its internal circadian timer which is longer than 24 h. It is synchronized to 24 h circadian rhythm by the environmental photoperiod clues. However, melatonin is a major chemical message to synchronize its activity of SCN [88].

Melatonin membrane receptors have been identified in the SCN of vertebrates [56,89] and the signal transduction pathways seemed to be involved in both MT1 and MT2 to induce an increase in the expression of two clock genes, Period 1 (Per1) and Period 2 (Per2) [89,90,91]. Without the feedback information of melatonin, SCN would not properly interpret the natural photoperiodic changes [92] and would exhibit a free running internal rhythm in which the cycle is longer than 24 h. In this situation the SCN would also instruct the pineal gland to exhibit an unusual melatonin circadian rhythm which is also longer than 24 h. This phenomenon is apparent in completely blind animals and humans whose eyes, specifically the MRGC, do not appropriately receive environmental photoperiodic information [93,94,95]. Importantly, melatonin administration to blind subjects partially re-entrains their biological rhythms close to normal [94,96,97].

Pineal gland is mainly comprised of pinealocytes, microglia and astrocytes. The lineage of pinealocytes is elusive. Current information suggests that pinealocytes are differentiated from Pax6-expresssing neuroepithelial cells [98]. They are specialized to synthesize and release melatonin (and possible some other substances). This explains why pinealocytes with two special characteristics regarding their mitochondria. First, the pinealocytes contain many more mitochondria than those of neuronal cells. Second, the morphologies of these mitochondria exhibit obvious dynamic alterations related to their fission, fusion and mitophagy activities during a 24 h period [99]. Because of the high density of mitochondria, we speculated the mitochondria are the major sites for melatonin synthesis [100]. Subsequent studies have proven this speculation. Melatonin synthesis was identified in the mitochondria of both animal and plant cells [101,102]. Recently, this was further confirmed by Suofa et al. [55]. They observed that the mitochondria are the exclusive sites of melatonin production in pinelocytes and in neuronal cells. The exact subsite of melatonin synthesis occurred in the matrix of mitochondria. Thus, the numerous mitochondria in pinealocytes relate to their melatonin synthetic function. This does not naturally exclude the extra-mitochondrial melatonin production. In cytosol, melatonin can also be synthesized. For example, red blood cells and platelets which are without mitochondria still produced melatonin [42,43]. Due to the substrate, particularly acetyl coenzyme A availability, melatonin synthesis in the extra-mitochondrial sites would not be as efficient as in the mitochondria since acetyl coenzyme A is concentrated in the mitochondria [99].

As to the mitochondrial dynamic alterations, generally, at darkness when melatonin is at its synthetic peak, more mitochondrial fusion was observed and, during the day, more fission was obvious. It was speculated that the mitochondrial dynamic changes were associated with their function, i.e., to produce melatonin [103]. However, current studies have reported that melatonin *per se* can regulate mitochondrial morphology [104,105]. Melatonin upregulates the levels of mitochondrial fusion proteins mitofusin 1 (Mfn1) and Opa1 to promote mitochondrial fusion [106,107] and inhibits the nuclear translocation of dynamin-related protein 1 (DrP1). The nuclear translocation of DrP1 increases mitochondrial fission and the inhibition of DrP1 nuclear translocation by melatonin results in suppression of mitochondrial fission [108,109,110,111,112]. Thus, the net result of melatonin is to promote the mitochondrial fusion and to reduce mitochondrial fission.

The effects of melatonin on mitophagy are still elusive. Some reports document that melatonin inhibits mitophagy and others show that melatonin promotes this process depending on the experimental conditions and cell type [109,113,114,115,116,117,118,119,120,121]. Currently it is not possible to determine whether the mitochondrial dynamic changes in pinealocytes relate to their functional activity which may be controlled by the clock genes, such as perd1, 2 or a result of their melatonin production rhythm. Thus, do the changes in melatonin levels generated by pinealocytes result in the mitochondrial dynamic changes.

In addition to the pinealocytes, the astrocytes and the microglia in the pineal gland also have the capacity to synthesize melatonin with great efficiency. The melatonin synthetic machinery including *AANAT*/*SNAT* and *HIOMT*/*ASMT* has been identified and melatonin production has been detected in these cells [45,122]. Markus et al. [123] hypothesized that melatonin synthesis was coordinated by both pinealocytes and macrophages/glia and astrocytes for the immunoresponse. For example, an acute inflammatory response drives the transcription factor, NFκB, to switch melatonin synthesis from pinealocytes to macrophages/microglia and, upon acute inflammatory resolution, back to pinealocytes. A participation of melatonin production by these cells would significantly improve the capacity of pineal gland to generate melatonin as a whole. This is particularly important in the situations in which melatonin is required, for example under the oxidative stress or inflammation. It was reported that CSF melatonin and its oxidative metabolites, AFMK, are elevated by several orders of magnitude in patients with meningitis [124]. This is probably the outcome of a coordinated physiology of all of the cells mentioned above. However, the major function of astrocytes and the microglia in the pineal gland is to regulate the pinealocyte melatonin synthesis under normal conditions. The regulatory mechanisms are well documented, i.e., astrocytes/glia are excited under different conditions which include elevated intracellular calcium concentration, which results in the NF-𝜅B activation. The excited astrocytes/microglia, then, release soluble TNFα which is the signaling molecule to the pinealocytes for inhibition of melatonin synthesis by targeting *AANAT* [98,125,126]. Other regulatory mechanisms may also be involved. For example, purinergic signaling on melatonin synthesis in pineal gland was reported [127]. ATP binding to its receptor in pinealocytes inhibits melatonin synthesis via suppression of the gene expression as well as the activity of the *ASMT* rather than the *AANAT*.

Once melatonin is synthesized in the pineal gland, it is rapidly released. The outlets for melatonin release are several. The classic concept is that pineal melatonin is released into the precapillary spaces, enters the capillaries, and then, via surrounding veins and sinuses reaches the general circulation. However, a more important route for melatonin release from pineal gland was uncovered, i.e., melatonin is directly release to the CSF of the third ventricle of the mammals. Compelling evidence supports this secretory route. Anatomically, a portion of pineal gland is nakedly-exposed into the CSF of the third ventricle (bathed by the CSF) [128] and many canaliculi of the pineal gland directly open into the CSF of third ventricle [129,130,131,132]. Pineal melatonin via these canaliculi is directly discharge into the CSF. This results in extremely high melatonin level in the CSF of pineal recess of third ventricle. In the sheep, the melatonin levels in the CSF of pineal recess of third ventricle are several orders of magnitude higher than those in the blood [133,134]. A melatonin concentration gradient in the CSF around the pineal recess of third ventricle was observed [135]. This indicated that the main source of CSF melatonin originated from the pineal recess of the third ventricle. The high melatonin levels in CSF have been reported in different species [136]. It is obvious that there are, at least, two parallel melatonin secretory routes, i.e., the general circulation and the CSF (Figure 3). The key question is which one probably transduces the photoperiodic information of retinas to the SCN-pineal loop, particularly to the SCN. If, as previously predicted, the CSF melatonin was from the blood, there was no doubt that general circulatory melatonin was the signal. However, it is known that the CSF melatonin is not from the blood but it is directly derived from the pineal gland; thus, the blood melatonin circadian rhythm as the signal of natural photoperiodic information is open to question, at least in terms of the major signal. Based on the evidence, it was hypothesized that the CSF melatonin released by pineal gland rather than the blood melatonin served as the signal of the natural photoperiodic information [135,137,138]. SCN is close to the third ventricle and the high levels of CSF melatonin can easily be transported into the SCN via simple diffusion or via tanycytes; these cells possess basal processes for the transport of small molecules including melatonin and this melatonin directly targets the SCN as the signaling molecule [130].

**Figure 2:**
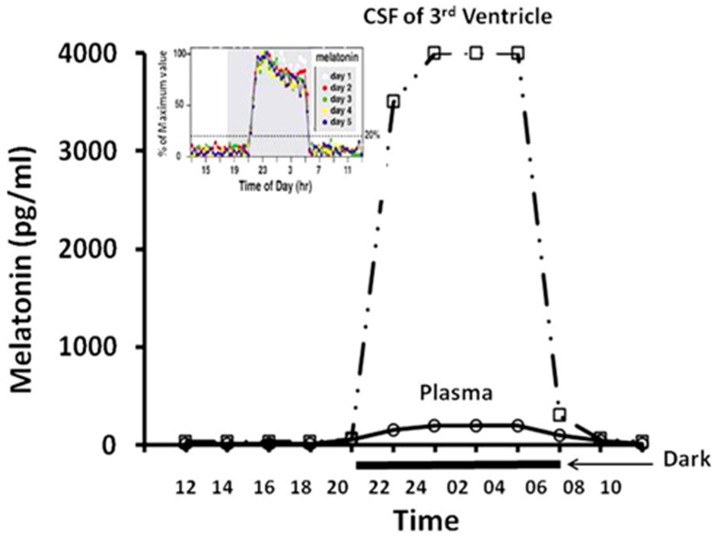
The different levels and shapes of melatonin circadian rhythms in the CSF of the third ventricle and in the peripheral blood. The nightly melatonin levels in CSF of the third ventricle are more robust than those in the peripheral plasma and also exhibit sharp rises and falls (square wave). The pattern of the melatonin circadian rhythm in CSF of the third ventricle is similar to that of the pineal gland rather than in the plasma (see the insert part which illustrates the melatonin synthetic pattern in pineal gland of rat). The data was obtained from the long term (5 days) pineal gland dialysis in a free running rat. Extrapineal-generated melatonin and the diet-derived melatonin may increase peripheral plasma melatonin levels; however, they do not mimic the pattern and reach the high level of the melatonin circadian rhythm in the CSF of the third ventricle to impact the function of bio-clock. From Tan et al. [63].

It is now a common knowledge that many foods contain melatonin. These include herbs, vegetables, fruits, cereals, beans, eggs, meets, fish, milk, wine, beer and coffee [139,140,141,142,143,144]. Consumption of these foodstuffs increases the circulating melatonin levels [145]. In some cases, the food-derived melatonin could elevate serum melatonin levels as high as the night time peak levels of melatonin [146,147,148,149]. Whether this food-derived melatonin alters the signaling information and produces chronobiological consequences remains unknown. If the melatonin in general circulatory system serves as the photoperiodic signaling; food-derived melatonin may have the chronobiological effects. If the CSF melatonin serves as the exclusive signaling to the SCN, the serum melatonin derived from food would not impact the chronobiology since the food-derived melatonin unlikely reaches night time CSF melatonin levels. The SCN is likely regulated by the high levels and square shape of melatonin rhythm that is completely different from the serum melatonin as to their shapes and it would not response to the low level and other shapes of melatonin rhythm input [63] (Figure 2). Thus, the photoperiod induced melatonin message is a precise trait that would not be influenced by non-CSF melatonin level alterations.

The high levels of CNS melatonin also exhibit protective effects on the brain tissue [130]. Pinealectomy results in the accelerated neurodegenerative changes and evidence of premature aging in animals [150,151,152,153]. Pineal grafts in brain protected the brain from oxidative damage induced by the ischemia/reperfusion in mice [154]. These effects are mainly attributed to the antioxidative, anti-inflammatory and anti-apoptotic effects of CSF melatonin directly released by pineal gland.

## 3. Pineal Gland Calcification (PGC), Melatonin Production, Neurodegenerative Diseases and Aging

Pineal calcification (synonyms include corpora arenacea, acervuli, brain sand, psammoma bodies and pineal concretions) was observed as early as in 1653 in humans [155]. Its presence was identified in a wide range of species including human, ox, sheep, horse, donkey, monkey, cow, gerbil, rat, guinea pig, chicken and turkey [156]. Even through the concretions were not found in the pineal organs of fish, amphibians, reptiles the high calcium content was detected in their pineal by ultrastructural calcium histochemistry [157]. Thus, pineal calcium metabolism and pineal calcification are wide spread phenomenon across species. Its rate increases with aging and in some species the pineal calcification rates are as high as 100% with age [158,159]. Ironically, calcification also occurs in neonatal humans [160,161]. It was reported that the pineal calcification in humans failed to impact the melatonin production and its circadian rhythm [77,78]. As a result, some believed that the pineal calcification might be a physiological process and not associated with pathological or aging changes. Rather, it might be related to the metabolic activity of pineal gland *per se*. In gerbils, the accumulation of pineal calcium deposits is blocked by superior cervical ganglionectomy which is believed to shut down completely the function of the pineal gland [162,163]. Gerbils exposed to short photoperiod (LD 10:14) exhibited significantly higher numbers of pineal concretions than those that were exposed to long photoperiod (LD 14:10) [164]. In addition, pineal calcification was enhanced in the gerbils with bilateral optic enucleation in which the animals are completely devoid a photoperiodic influence [165] with the generation of more melatonin. This evidence supported the metabolic theory of pineal calcification.

Large amounts of evidence, however, also suggest that the pineal calcification was indeed associated with human pathological disorders and aging. Decades ago several studies pointed out the relationship between the pineal calcification and schizophrenia [73,166,167,168]. The highest pineal calcium content was detected in the pineal gland of patients who died of renal disease associated with hypertension among other diseases [169]. Currently, additional studies have reported the strong association of PGC and neurodegenerative diseases, particularly Alzheimer’s disease [170]. This association is connected with the melatonin levels synthesized by this gland. It is well established that melatonin is a neuroprotector with its potent antioxidant function and anti-inflammatory activity [171,172,173,174,175,176]. The brain is rich in lipid, lacks the antioxidative enzyme, catalase, and consumes large quantity of oxygen (roughly 20% of the total oxygen consumed by the brain with 1% of the total body weight). This makes the brain more vulnerable to the oxidative stress than other organs. Decrease of endogenous melatonin will result in the neurons being less resistance to the oxidative stress or brain inflammation. Several studies have reported the negative association between the Alzheimer’s disease and serum or CSF melatonin levels [177,178]. The mechanistic investigations uncovered that in addition to its antioxidant and anti-inflammatory activities, melatonin directly inhibits the secretion and deposition of the β amyloid protein (AD plague) [179,180] which is the hallmark of this disease; it also suppresses tau protein hyperphosphorylation thereby reducing intracellular neurotangles [181,182,183], another biomarker of AD. The majority of the small scale clinical trials support that melatonin application improved the symptoms of sundowning syndrome and retarded the progress of AD [184,185,186,187,188,189,190].

The most suggestive results come from the animal studies. In single, double or triple gene mutated AD animal models large doses of melatonin (100 mg/L drinking water or 10 mg/kg body weight/day) prolonged their life span, positively modulated the biochemical and morphological alterations and improved their cognitive performance [191,192,193,194,195,196]. To date, the large doses of melatonin used in animal studies have not been applied in clinical trials of Alzheimer’s disease. Considering the unique safety margin of melatonin, larges dose of melatonin can be used in AD patients and it may achieve its maximum treatment effects on this devastating disease. Recently, it was found that melatonin treatment for the sporadic AD animal model (OXYS rats) also produced impressive results. Very interestingly, OXYS rats exhibit significantly lower endogenous melatonin levels during night compared to their controls (Wistar rats). Melatonin treatment was especially effective in preserving the microstructures of hippocampal neurons and their mitochondrial distribution and integrity in this pathological animal model [197,198,199,200]. The sporadic AD includes roughly 95% of the clinical AD cases. These observations provide solid evidence suggesting the use of relatively large doses of melatonin to treat AD clinically. In a few cases, melatonin treatment did not result in expected results in AD patients [201] or in animals [202]; however, there were, at least, no serious adverse effects of the treatment. For other neurodegenerative diseases including Parkinson’s disease, amyotrophic lateral sclerosis (ALS), multiple Sclerosis (MS) and Huntington’s disease, melatonin applications also achieved positive results in patients and animal models [203,204,205,206,207,208]. For example, in MS patients their biochemical markers and some of the symptoms were improved after melatonin supplementation [209,210,211,212,213,214].

As to the association between the aging and melatonin production, in most vertebrates, melatonin production wanes with aging. The reasons for this may be two-fold. Melatonin synthetic capacity is dampened during aging due to the reduced density of β-adrenergic receptors in the pineal gland [215,216] and the downregulation of gene expression or phosphorylation of *AANAT*/*SNAT* [217]. A second reason is the increased consumption of melatonin. This is due to the metabolic alterations. For example, more ROS are generated by the aged cells than in the young cells and melatonin as the endogenous antioxidant is used to neutralize the overproduced ROS in aging organisms. Both of these effects may cause its low levels in the aged vertebrates. Low melatonin level is considered as a biomarker of aging [218,219,220]. When melatonin production was depressed by pinealectomy in rats, accumulation of oxidatively-damaged products accelerated their aging process [221]. In contrast, when young pineal glands were grafted to the old animals or exogenous melatonin was supplemented, both significantly increased the life span of experimental animals [222].

A great deal of attention has recently been given to the relationship of decreased melatonin levels in neurodegenerative diseases and aging associated pineal calcification. With the increased use of the PET scan, susceptibility-weighted magnetic resonance imaging (SWMR) or other advanced technologies, even very small pineal concretions can be identified in patients or animals, which could not be seen previously. It was found that the rates of pineal calcification have been significantly underestimated previously. For example, in non-specifically targeted patients with the average age of 58.7 ± 17.4 years, 214 out of 346 showed PGC on CT scans (62%) [223]; the data of 12,000 healthy subjects from Turkey indicated that the highest intracranial calcifications occurred in the pineal gland with an incidence of 71.6% [65]. PGC appears to occur without significant differences among countries, regions and races. For example, in Iran the PGC incidence is around 71% [224] and in African (Ethiopia), it is roughly 72% [225] and in black people in the US it is 70% [226]. With such a high incidence of PGC in humans and considering the functions of pineal gland, the PGC should not be considered a normal physiological process.

PGC is often related to the decreased melatonin levels and several pathological alterations including neurodegenerative diseases (Alzheimer’s, MS), migraine, symptomatic intracerebral hemorrhage, symptomatic cerebral infarction, sleep disorders, defective sense of direction and pediatric primary brain tumor [73,170,227,228,229,230,231]. Interestingly, PGC is mainly associated with brain-related disorders but not few with other organ pathophysiologies while the decreased melatonin levels were detected in the blood which supplies all the tissues. This observation further supports our hypothesis that high levels of melatonin released directly into CSF from pineal gland serve as the biological circadian rhythm regulator and the neuronal antioxidant while the blood melatonin is the residue of the pineal melatonin [63,130]. This residual melatonin only resembles the CSF melatonin rhythm and may be without significant biological functions.

PGC reduces CSF melatonin levels and dampens its rhythm resulting in chronological disturbance including insomnia and migraine. The low levels of CSF melatonin also elevate neuronal damage from ROS, thus, accelerating the neurodegenerative disorders.

It also has been reported that the serum and salivary melatonin and its urine metabolite are negatively related to the size of the pineal calcification and positively related to the uncalcified portion of the gland [232,233,234,235]. In Alzheimer’s disease, the patients had a higher portion of calcified pineal glands and lower portion of uncalcified glands than patients with other dementias [170]. As mentioned, that the serum melatonin rhythm resembles the CSF melatonin; thus, it can be deduced that the CSF melatonin levels in Alzheimer’s patients would also be significantly reduced. It is difficult to obtain CSF from the Alzheimer’s patients to test melatonin levels. However, the postmortem CSF from these patients indeed proved the low levels of melatonin. Their CSF melatonin level was only 20% of that in their non-Alzheimer’s controls. The authors suggested that the reduction in CSF melatonin levels might be an early event in the development of AD possibly occurring even before the clinical symptoms [177,178]. If these patients had PGC, their CFS melatonin level may further decrease and it would accelerate the process of the disease. The PGC is also associated with aging [236,237] even through the PGC has been detected in the neonatal or in children. It was reported that the incidence of the visible PGC increases with age, i.e., 2% at 0–9, 32% at 10–19, 53% at 20–29 and 83% in over-30 age groups, respectively [238]; clearly the degree of PGC increased with aging [239]. In turkeys and rats the incidence of PGC reaches 100% in advanced age animals. It seems that PGC is an inevitable process of aging in vertebrates. If so, slowdown of this process may retard the aging process. This is discussed later.

## 4. Potential Mechanisms for PGC Formation

Even through PGC is a widespread phenomenon in vertebrates, its importance has been ignored and little attention has been given to this important issue for decades. To date, little is known about its exact formation processes and mechanisms. Here, we summarize several opinions and speculations on the potential mechanisms of PGC formation and also discuss our hypothesis regarding these enigmatic structures. It seems that there are two origins of PGC, that is, in association with pinealocytes or with non-pinealocytes. Some studies found that PGC was restricted to the connective tissue. The mechanisms involved the formation of calcareous deposits within the connective tissue stroma of the gland [240]. These deposits represent the aging-related calcium accumulation within the connective tissue. This type of calcification is similar to that found in the habenular commissure and choroid plexus [238]. The connective tissue derived PGC is predominant in the rat and Pirbright white guinea pig [241,242]. In analysis of the specimens of human pineal gland, Maslinska et al. [160] reported that the initiation of PGC was associated with the tryptase-containing mast cells. During the systemic or local pathological conditions, the tryptase-containing mast cells infiltrate into the pineal gland where they release biologically active substances including tryptase which participates in calcification. This process is pathological but not age related since it also occurs in the children.

**Figure 3:**
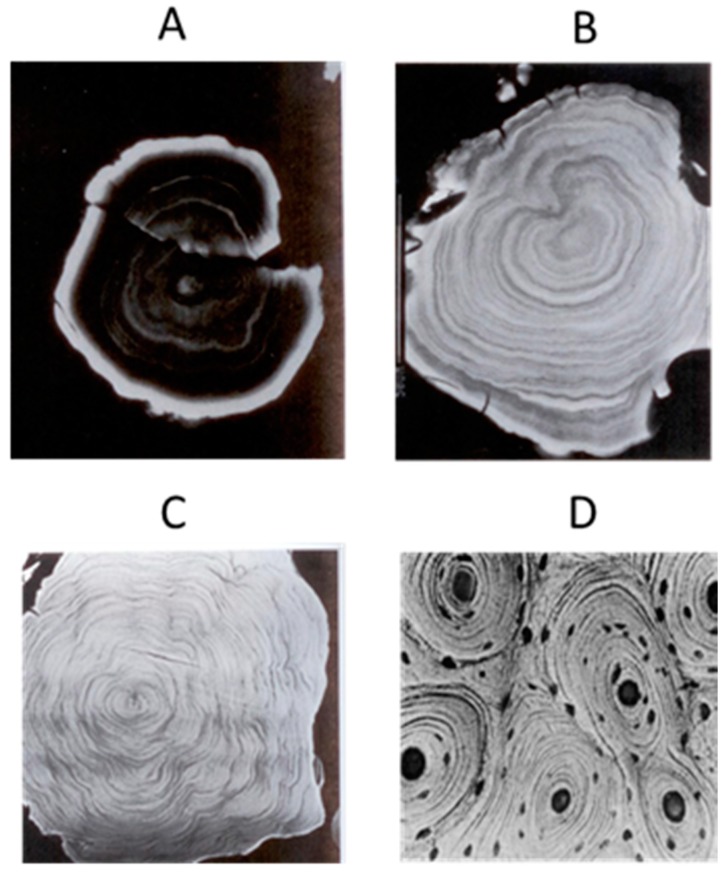
The laminated pineal gland calcification at different ages and their similarity to the osteons of compact bone. (**A**) Pineal calcification in 14 year old subject; (**B**) 47 year old; (**C**) 62 year old; (**D**) osteons; (**A**–**C**) were modified from Hermann et al. [239].

As to the PGC of pinealocyte-origin, two speculations should be mentioned. One is proposed by Lukaszyk and Reiter [13,243]. They reported that the pinealocytes extruded polypeptides into the extracellular space in conjunction with their hypothetic carrier protein, neuroepiphysin. The pineal polypeptides of exocytotic microvesicles were actively exchanged for the calcium. The calcium-carrier complex then is formed and deposited on the surface of adjacent mutilayed concretions. Thus, the concretion formation is related to the secretory function of pineal gland. For example, in the gerbil following the superior cervical ganglionectomy, the PGC are completely inhibited; this was attributed to a decrease in the functional activity of the gland [163]. However, this cannot explain the observation of intracellular calcification of the pinealocytes [244,245]. Krstić [246,247] proposed another mechanism to explain the origin of PGC from pinealocytes. He speculated that the cytoplasmic matrix, vacuoles, mitochondria and the endoplasmic reticulum of large clear pinealocytes were the initial intracellular calcification sites. These loci, and particularly those within the cytoplasmic matrix, transformed into acervuli by a further addition of hydroxyapatite crystals. The cells gradually degenerated, died, broke down, and the acervuli reached the extracellular space. High intracellular calcium levels could be a situation that is responsible for eliminating calcium from the cell, with the hypercalcemic intracellular milieu promoting the initial crystallization. The failure of Ca^2+^-ATPase could be a natural process of aging or pathological conditions [248]. Hence, PGC does not occur under normal conditions and it is a result of altered molecular processes in vertebrates. These speculations; however, cannot completely explain the mechanisms of the PGC formation. Here, we provide an additional speculation which is a complementary of the previous suggestions. It seems that the PGC in some cases is an active rather than a passive process. We previously hypothesized that the pineal gland may have a blood filtration function like the kidney since its vascular structures as well as its blood flow rate are similar to the kidney [63]. The question is whether they share a similarity to the calcification this is observed in both organs. It is well documented that the compositions of PGC is totally different from the kidney stone. Kidney stones are primarily composed of calcium oxalate and its formation is simply a sedimentary process caused by high concentrations of both calcium and oxalate [249]. A main component of a PGC is hydroxyapatite [Ca_10_(PO_4_)6(OH)_2_] [248,250,251] which is the chief structural element of vertebrate bone. The Ca/P molar ratio in pineal concretions is similar to the enamel and dentine [252] and these authors pointed out that the nature and crystallinity of the inorganic tissue of the pineal concretions lead one to think of a physiological rather than pathological ossification type with characteristics between enamel and dentine. It is not very clear how the hydroxyapatite is formed in the bone but there is little doubt that its formation involves the collaboration of bone cells and it is a programmed process. In addition, the concentric laminated pineal concretions are frequent observed [157,239] to be structurally similar the osteons (Figure 3), the major unit of compact bone.

**Figure 4:**
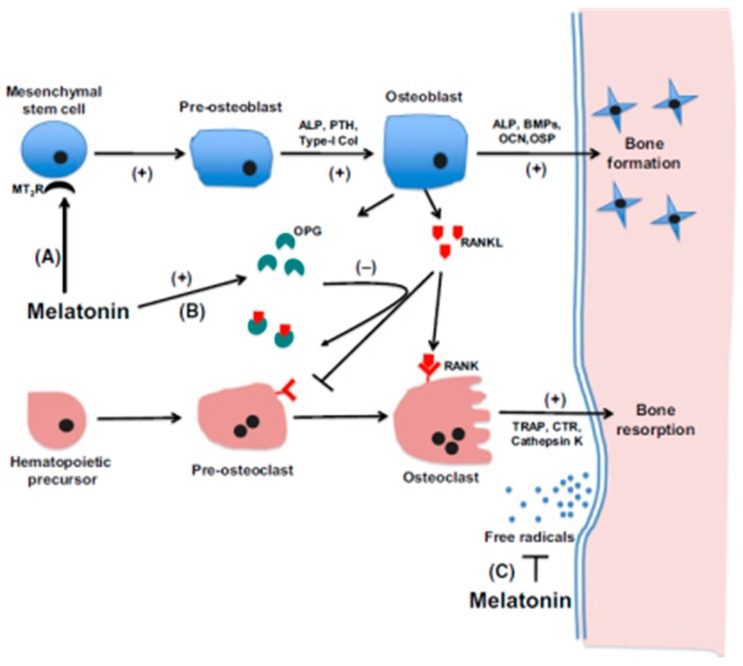
The proposed mechanisms underlying melatonin’s actions on bone formation. (**A**) melatonin induces MSCs differentiation into osteoblasts via MT2; (**B**) it promotes osteoprotegerin (OPG) expression in preosteoblasts which would inactive RANKL, leading to a suppression of osteoclastogenesis; and (**C**) through melatonin’s free-radical scavenging and antioxidant properties, protecting against radical induced loss of osteoblasts and osteoclasts. PTH (parathyroid hormone); Type I col (type I collagen); OSP (osteopontin); BMP-2 (bone morphogenetic protein 2); ALP (alkaline phosphatase); OCN (osteocalcin); TRAP (tartrate-resistant acid phosphatase); RANKL (receptor activator of NFĸB ligand); OPG (osteoprotegerin). From Maria and Witt-Enderby [262].

The laminated pineal stone indicates its formation is not random but organized and programmed. For example, in humans, laminated pineal stones are associated with aging. The older the individual, the larger number of lamellae (Figure 3) [239]. Our hypothesis is that the pineal calcification, at least partially, may be similar to the bone formation that is, the pineal calcium deposit may be formed by differentiated bone cells under certain conditions. Recently, numerous studies have reported that melatonin facilitates the capacity of mesenchymal stem cells (MSCs) to differentiate into osteoblast-like cells under in vivo or in vitro conditions [253,254,255,256,257]. Mesenchymal cells are found in the early stage of pineal development in birds and in rats [258,259]. Mesenchymal cells have an important role in pineal follicular formation later during development of the gland. It was also documented that the striated muscle fibers are present in the pig and rat pineal gland [260,261]. These striated muscle fibers are of mesenchymal rather than ectodermal origin [261]. These observations indicate that the MSCs are present in the pineal gland and they have the capacity to differentiate into different cell types including muscle as well as probably the osteoblasts and even the osteocytes. The MSCs in the pineal gland may be retained from its early embryonic stage of mesenchymal tissue and/or they may be of vasculature origin. The differentiation from MSCs into osteoblasts/osteocytes seems to be melatonin dependent. The signal transduction pathway of this transition is probably mediated by melatonin membrane receptor 2 (MT2) [261]. The detailed mechanism was proposed by Maria and Witt-Enderby [262]. Simply, melatonin binds to the MT2 of MSCs to promote them to differentiate into pre-osteoblasts. At the same time melatonin increases the levels of parathyroid hormone (PTH); type I collagen and alkaline phosphatase (ALP) and these factors further promote pre-osteoblasts to form osteoblasts. Finally, melatonin upregulates the gene expression of the osteopontin (OSP), bone morphogenetic protein 2 (BMP-2), osteocalcin (OCN) and ALP and facilitates the osteoblast proliferation, osteocyte formation, mineralization and bone formation (Figure 4.)

**Figure 5:**
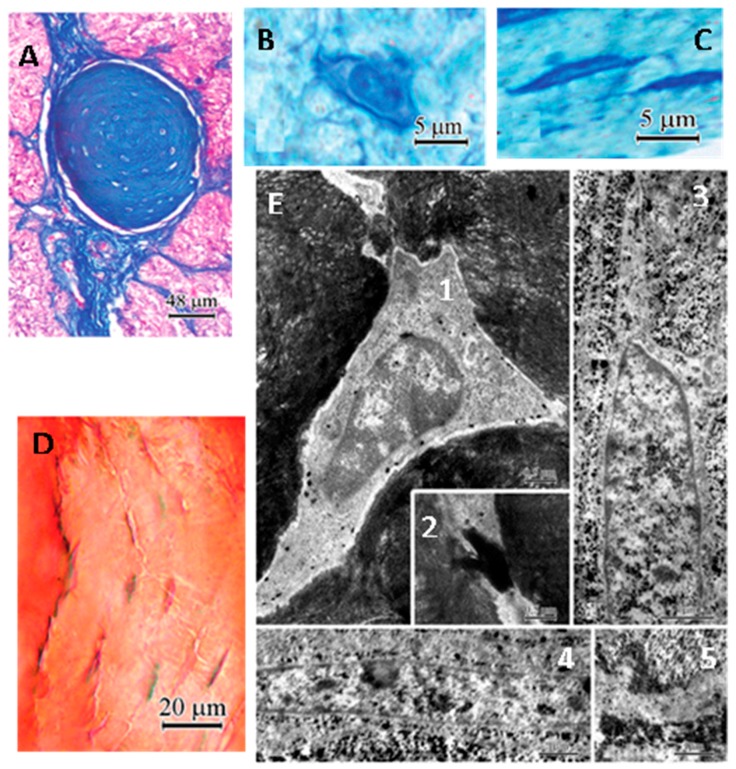
Histology and ultrastructure of cells located in a concretion of the turkey pineal gland. (**A**) mature concretions identified with Mallory’s stain. Two types of cells are present in the concretion (semi-thin section stained with toluidine blue); (**B**) polygonal cells; (**C**) elongated cells; (**D**) The presence of calcium in the concretions was demonstrated with Alizarin red S; note the characteristic appearance of cells located in the concretion; (**E**) ultrastructure of cells located in the calcified area (fixation with the PPA method). 1: the osteocyte-like cell surrounded by mineralized collagen fibrils in the central part of the calcification area. Note the “halo” around the cell and large pyroantimonate precipitate located mainly outside the cell membrane, 2: the junction of processes of osteocyte-like cells, 3: a cell showing a fibrocyte-like appearance in the peripheral part of the calcification area. Note the extra-cellular matrix containing collagen and calcium deposits, 4: numerous calcium precipitates in the intercellular spaces in the peripheral part of the calcification area, 5: The cell process with scattered deposits in the middle part of the concretion. Note the adjacent extra-cellular matrix rich in collagen and pyroantimonate precipitates. Modified from Przybylska-Gornowicz et al. [159].

MT2 has been identified in MSCs using molecular techniques and classical pharmacology [263,264]. Transgenic knockout of the MT2 in mice inhibited the osteoblast proliferation and bone formation [265]. This indicates that the pineal gland has the capacity to form the bone like structure (calcification) by the pathway including MSCs. The promotor is the high levels of melatonin generated by this gland. The process of PGC in bird (turkey) resembles the bone formation which strongly supports our hypothesis. It requires a microenvironment which includes collagen fibrils, phosphate and calcium. The osteocyte-like cells are found in the center of the pineal concretion and the peripheral part contains the osteoblast-like cells and densely packed collagen fibrils [159] (Figure 5). The intermediate portion is the place of mineralization as bone.

Based on the current knowledge, we speculated that the “osteocyte-like cells” and the “osteoblast-like cells” were osteocytes and osteoblasts which differentiated from the MSCs in the pineal gland under the influence of melatonin. If the pineal microenvironment facilitates the PGC formation, why are the PGC often associated with aging and some pathological conditions?

Currently, we cannot definitely answer this question, but several clues might indicate the relationship of PGC with aging/pathology:

(1) *Chronic vascular inflammation*: The pineal gland has a complicated vascular system with abundance of arteries, fenestrated capillaries and veins. Especially the filtration rate of blood in pineal gland is in excess of most organs and it is only second to the kidney in terms of blood flow. These make the gland venerable to the chronic vascular inflammation during aging or certain disorders. The vascular inflammation mobilizes the MSCs migration and adhesion in the gland or promotes the de novo MSCs proliferation due to the increased levels of pro-inflammatory cytokines, TGF-β or TNF-α. The crosstalk between vascular MSCs and inflammatory mediators, especially, interleukin-22, lead to MSCs proliferation, migration and osteogenic differentiation [266,267] under the influence of high levels of pineal melatonin and finally PGC formation.
(2) *Brain tissue hypoxia*: Many pathological conditions cause brain tissue hypoxia including hypertension, sleep apnea, stroke, and even respiratory disorders. Hypoxia-inducible factor (HIF)-1α is an important regulator of MSCs and it promotes the proliferation, migration and adhesion of MSCs in the hypoxic areas [268,269,270] including to the pineal gland. Generally, hypoxia increases bone resorption and suppresses osteoblastic differentiation and bone-formation [271,272]. However, this may not be applied to the pineal gland. During the dark phase, the pineal produces high levels of melatonin. Under the hypoxic condition, melatonin would promote the osteoblast differentiation and mineralization of MSCs via the p38 MAPK and PRKD1 signaling pathways [273]. In addition, melatonin also inhibits the activity of the osteoclast and osteoclatogenesis [274,275], especially under inflammatory conditions [276]. These processes favor PGC formation under hypoxic conditions.
(3) *Intracranial pressure*: Some cells of the pineal gland are “swimming” in the third ventricle and, as a result, they are influenced by the intracranial pressure. Intracranial pressure usually increases with cerebral disorders such as idiopathic intracranial hypertension, brain trauma and stroke [277], and even Alzheimer’s disease [278]. The high pressure may impede the pineal filtration rate and induce endoepithelial cell damage by chrono-inflammation. The pressure also promotes the bone remodeling and mineralization, thus, PGC formation.

## 5. Rejuvenation of Pineal Gland?

As mentioned, the pineal gland may be an important organ for maintaining the optimal health of vertebrates. Its malfunctions including its calcification may have associations with the premature aging and aging-related diseases. To answer this question, researchers had tried to rejuvenate the gland by ectopically pineal transplantation. Initially, it was found that the pineal glands which were transplanted into the anterior chamber of the eye in rats were innervated by surrounding sympathetic nerve endings and their normal rhythm in *AANAT* activity was established similar to the in situ pineal gland [279,280]. To support this observation, more complicated studies have been performed in which pineal glands were transplanted into a variety of sites in pinealectomized rats. These sites included anterior chamber of the eye, third cerebral ventricle, the pineal region (in situ transplantation), intrastriatal, renal capsule and thymus [280,281,282,283,284,285]. The results indicated that in some cases the pineal gland transplantation did increase the melatonin levels in pinealectomized animals; however, except in the site of anterior chamber of the eye, no melatonin circadian rhythm was detected with the pineal gland transplantation and also the melatonin levels could not match those of the in situ pineal gland produced. The lack of melatonin rhythm after pineal transplantation may relate to lack of sympathetic innervation of the grafted gland at other sites as compared to the anterior chamber of the eye in which the sympathetic innervation was obvious [286].

In addition to the anatomic and morphological studies, the functional studies of ectopic pineal gland grafts provided promising results. When the pineals of young mice (3–4 months) were grafted into thymus of the old mice (17–18 months), they partially prevented thymic involution in the old animals due to an anti-apoptotic activity [287]. The similar result was observed in the rats. When the pineal glands of young animals were transplanted into the thymus of old rats, they preserved the age-related alterations in erythrocyte membranes by increasing their hemolysis time and decreasing their peroxidation [288]. The young pineal transplants into the thymus of old mice could even prolong the recipients’ life span up to 27% compared to the controls [222]. The authors attributed the life prolongation effects to that the grafted pineal gland might release high level of nocturnal melatonin which acted on the thymus to rejuvenate the gland and preserved the immune responsiveness of these old animals to the levels of young animals. Similarly, the intrastriatal transplantation of pineal tissue significantly reduced the brain infarct size in middle cerebral artery occlusion (MCA) ischemia/reperfusion animal model [154].

In general, ectopic pineal gland transplantation appears to be beneficial for health in many aspects. However, it is obvious that it cannot replace the function of the in situ pineal gland. As mentioned previously, the in situ pineal gland produces high level of melatonin, which protects the brain from the oxidative damage after its release into the CSF; secondly, pineal melatonin secretion exhibits a circadian rhythm, especially in the CSF of the third ventricle in which the night time melatonin peak have a sharp rise and fall compared to its serum circadian pattern (Figure 2). This CSF melatonin alteration is believed to serve as the signal of biorhythm of organisms [289]. The ectopic pineal gland transplants lack these two most important aspects. Thus, an improved means to mimic activities of the in situ pineal gland would probably be the pineal transplantation into the pineal region (in situ graft) or into the third ventricle. Some studies have been performed in this regard. The results indicate that the pineal glands which were transplanted into third ventricle or pineal region (in situ transplantation) survived due to re-vascularization and partial re-innervation [7]. They did produce melatonin, but the levels were low and completely without the night time rise [7,283].

Based on these results, we speculated that a more suitable way to preserve a healthy and functional pineal gland is either to retard its calcification or to recover the functions of the calcified gland. As mentioned, several pathological conditions might promote the premature pineal calcification. However, the environmental biohazards may also contribute to its development. One of them is fluoride. It was reported that the pineal gland in goosander concentrates fluoride which is a water pollutant [290]. The level of fluoride in the pineal gland of goosander was 5-fold higher than that it in the brain of the animal. The similar results were observed in the aged human pineal gland. In addition, the high level of fluoride in the human pineal gland is positively related to its calcium accumulation of the gland [291]. Thus, decrease in environmental fluoride pollution may be helpful in delaying or avoiding premature pineal calcification. It was hypothesized that the lack of calcium salt crystallization inhibitors, such as pyrophosphate and phytate, would favor calcification [292]. Studies indicated that the phytate content in brains of healthy animals was 10-fold higher than that in other tissues [293,294]. Increases in the availability of calcium salt crystallization inhibitors would tend to protect against pathological pineal calcification.

Finally, the pineal gland decalcification may not impossible. Currently, pineal microdialysis is frequently used to measure the melatonin production of the pineal gland [295,296,297,298]. This method could also be used to decalcify the gland by use of EDTA or/and acidic solution as the eluent. This solution would have the ability to dissolve the calcium deposits and removed them by dialysis. Also, cells isolated from young pineal gland or engineering-modified stem pinealocytes could be directly injected into the decalcified in situ pineal gland. Such transplanted cells have the high chance of survival in the gland due to the melatonin level generated by the gland. It was frequently reported that elevated levels of melatonin effectively promotes the transplanted stem cell survival and differentiation in different organs and tissues [299,300,301,302,303]. In a preliminary study, we mixed 2 × 10^5^ cells (in 20 μL) collected from pineal gland of one day old chicks were injected into the in situ pineal gland of 4.5–5 year old hens. The results indicated that this procedure improved egg laying rate and the general wellbeing of the old recipients (unpublished observations). This is the first step to rejuvenate the calcified pineal gland to maintain optimal healthy status of humans.

## 6. Conclusions

Accumulating evidence indicates that pineal health is important to preserve the optimal physiological status of animals, including humans. The pineal gland is a unique organ which synthesizes melatonin as the signaling molecule of natural environmental changes and as a potent neuronal protective antioxidant. This gland undergoes calcification due to its anatomic structure (rich in vasculature and blood flow) and functions (melatonin production and CSF generation). The pineal has the highest calcification rate among all organs and tissues. Pineal calcification jeopardizes the melatonin synthetic capacity of this gland and is associated with a variety of neuronal diseases. Although PGC is found in neonates, its occurrence is primarily associated with pathological conditions and aging. The exact mechanisms of how it occurs are currently unknown; however, several theories have been proposed to explain calcium deposit in the gland. We hypothesize that PGC is an active process which is similar to bone formation, that is, the osteocytes (or osteocyte-like cells) and osteoblasts (or osteoblast-like cells) are involved. These cells probably differentiate from MCSc which are the *de nova* MCSc of the gland; alternatively, they migrated from the vasculature under pathological conditions such as chronic inflammation. High levels of melatonin generated by the gland promote PGC since this molecule enhances the differentiations of MCSc into osteoblasts and osteocytes. To compensate for the functional loss of the pineal gland, pineal grafts have been performed in different organs and tissues. The grafted pineal, however, cannot mimic the functions of the in situ pineal gland, especially since they do not establish the normal melatonin circadian rhythm. Thus, perhaps the best way to preserve a healthy pineal gland is to rejuvenate the in situ pineal gland by decalcification and then stem cell injection into the gland. It is speculated that a healthy pineal gland would be response to high level of melatonin production which benefits to immunomodulation, metabolic balance and anticancer effect generally [304,305,306]. Thus, this process and its outcomes should be investigated with enthusiasm in the future.
